# Predictors of postpartum depression in Syrian refugee women: indirect pathways between postmigration stress and depression through resilience and social support

**DOI:** 10.3389/fpubh.2025.1643089

**Published:** 2026-01-07

**Authors:** Taghreed N. Salameh, Ceren Acarturk, Shuying Sha, Lynne A. Hall, Seda Guney

**Affiliations:** 1 College of Nursing, QU Health Sector, Qatar University, Doha, Qatar; 2 Department of Psychology, Koç University, Istanbul, Türkiye; 3 School of Nursing, University of Louisville, Louisville, KY, United States; 4 School of Nursing, Koç University, İstanbul, Türkiye

**Keywords:** anxiety, intimate partner violence, postmigration stress, postpartum depression, posttraumatic stress disorder, refugee women, resilience, social support

## Abstract

**Background:**

Refugee women are at high risk for developing postpartum depression (PPD). This study aimed to examine the relationships of postmigration stress, intimate partner violence, anxiety, posttraumatic stress disorder (PTSD) symptoms, social support, and resilience with PPD, and to explore if social support and resilience serve as indirect pathways linking postmigration stress and PPD in Syrian refugee women in Türkiye.

**Methods:**

Data for this cross-sectional study were collected using structured telephone interviews from a convenience sample of 200 Syrian refugee women living in Türkiye between August 2022 and February 2023. Participants completed validated measures, including the Edinburgh Postnatal Depression Scale, Multidimensional Scale of Perceived Social Support, Connor-Davidson Resilience Scale, Posttraumatic Stress Disorder Checklist for DSM-5, Generalized Anxiety Disorder-7, Abuse Assessment Screen, and Postmigration Living Difficulties scale. Logistic regression analysis was used to examine the predictors of PPD. The bootstrapping technique with bias-corrected confidence intervals was employed to estimate indirect effects in Mplus.

**Results:**

The findings of this study revealed that resilience (adjusted odds ratio [AOR]: 0.921, 95% Confidence Interval [CI]: 0.867–0.979) and anxiety (AOR: 1.338, 95% CI: 1.17–1.53) significantly predicted PPD. Path analysis demonstrated that postmigration stress was indirectly associated with PPD through Social support (*β* = 0.033, 95% CI: 0.004–0.079) and resilience (*β* = 0.157, 95% CI: 0.077–0.244).

**Conclusion:**

Among the examined factors, only anxiety and resilience predicted PPD. Syrian refugee mothers in Türkiye might experience unique social life conditions that influence reporting postmigration stress and PTSD. Whereas social support and resilience served as significant indirect pathways between postmigration stress and PPD, the cross-sectional nature of the data precludes causal or temporal inferences. Yet, Interventions targeting anxiety and aimed at enhancing social support and resilience are suggested to reduce PPD symptoms among refugee women.

## Introduction

Postpartum depression (PPD) is prevalent among forcibly displaced women. It affects 25% of women who are refugees and asylum seekers (1). PPD often co-occurs with anxiety and posttraumatic stress disorder (PTSD) symptoms (2) and has negative health consequences that affect not only the mother but also her child and the whole family (3). However, more information about this significant mental health problem in refugee women is required worldwide (4), particularly in Türkiye, which hosts the highest number of refugees in the world (5). A better understanding of the determinants of PPD among refugee women is vital for healthcare providers to customize prevention and treatment interventions to their unique needs.

PPD is a cluster of depressive symptoms experienced in the first 12 months following childbirth (6). Whereas the psychosocial factors associated with PPD are well studied, there are few studies specifically targeting refugee women (1). Refugee women might have experienced a wide range of traumatic war-related violence and abuse (7). They may continue the experience of past trauma in the form of postmigration stress due to a lack of family and social network, social stigma, language barriers, and unemployment (8). A systematic review indicated that 21.4% of refugee or displaced women experienced sexual violence by an intimate partner or a stranger (9). Qualitative studies of Syrian refugee women settled in Jordan revealed that their experience of trauma from war and conflict and the disruption of their cultural norms led to intimate partner violence (10, 11). Although a review of Turkish newspapers (2015–2019) reported that Syrian refugee women experienced gender-based violence (12), there is limited information about intimate partner violence, particularly among postpartum women. Dennis et al. (13) found that postpartum migrant women in Canada, including refugees who experienced violence during pregnancy and/or post-birth, are at a higher risk of developing PPD. However, those who reported higher levels of social support are at a lower risk. This finding underscores the importance of social support for Syrian postpartum women, particularly in light of their cultural practices that emphasize the crucial role of female relatives in providing strong social support after childbirth (14).

Resilience plays a pivotal role in mental health, and understanding its dynamics among refugee women is crucial for enhancing cultural competence in policies and clinical practices (15). Resilience is a dynamic process in which the person manifests positive adjustment and adaptation in terms of interpersonal and social competence despite experiencing trauma and adversity (16). A qualitative study of mothers living in refugee camps in Türkiye and Syria revealed three coping resources: personal faith, reaching out for support, and adaptation to a new norm (17). However, there are sparse studies addressing resilience in postpartum refugee women and its influence on PPD. Therefore, this study aimed to: (a) examine the predictive relationships among postmigration stress, IPV, anxiety, PTSD symptoms, resilience, social support, and PPD in Syrian refugee women living in Türkiye, and (b) explore whether social support and resilience serve as indirect pathways linking postmigration stress and PPD.

### Theoretical framework

This study is based on the transactional model of stress (18). This theoretical model explains the relationship between stressful events and psychological outcomes. The structure of the transactional model imposes the influence that personal and situational factors have on the appraisal of a condition (i.e., primary and secondary appraisal) (18). The primary appraisal of a threat of loss or harm depends on a person’s antecedent conditions related to the threatening stimulus and the individual’s psychological characteristics. Lazarus and Folkman (18) label the outcomes of the primary appraisal as harm/loss, threat, and challenge. Hence, migration, being a refugee, and the delivery of a newborn may threaten the ability of a mother to enjoy or fulfill her expected roles and therefore influence her mental status (19). In addition, situational variables can affect cognitive appraisal; a more severe perception of the event would lead to a more intense appraisal and, in turn, a more stressful perception. In secondary appraisal, the person evaluates whether she/he has the competencies and resources, such as social support, to deal with stressors and restore equilibrium with the environment (18). In the transactional model, adaptational outcomes are considered as the product of the situational and personal variables. For instance, social support and resilience improve the interactions between personal and situational variables (i.e., postmigration stress) and have negative relationships with the adaptational outcome (i.e., postpartum depression).

## Methods

### Design and participants

This study used a cross-sectional design. Convenience sampling was used to recruit Syrian postpartum refugee women in Istanbul, where 532,000 Syrian refugees live (20), through referrals from the nongovernmental organization (NGO), Refugee and Asylum Seekers Assistance and Solidarity Association (RASASA). Refugee women who were aged 18 years and older and had a live birth within the past year were eligible to participate. Exclusion criteria included self-reported diagnosis of a major mental disorder (such as schizophrenia or psychoses) and current use of antipsychotic or antidepressant medications at the time of the study. Data were collected between August 2022 and February 2023.

### Sample size

The sample size was determined before participants were recruited. A minimum of 200 mothers was required for the path analysis in structural equation modeling, with a medium effect size of 0.30, a power of 0.80, and a significance level of 0.05 (21).

### Ethical consideration

This study was conducted per the ethical principles of the Declaration of Helsinki. Ethical approval was obtained from Koç University Committee on Human Research (Approval #: 2022.047. IRB3.019, Approval Date: 1/27/2022). Participants were informed about the study’s purpose and voluntary participation, including their right to decline or withdraw at any time without any influence on their care or services. Anonymity was assured, and participants were informed that their data would be used solely for research purposes. Eligible women who agreed to participate in the study provided recorded verbal consent. In addition, all participants were given information about available psychosocial services, including those offered by NGOs.

### Recruitment procedure

Following the referral of refugee women from the NGO to the primary researcher, a trained Arabic-speaking research assistant called each woman to explain the purpose of the study and review the eligibility criteria. Those eligible and who agreed to participate were asked to provide verbal consent to be contacted for a subsequent telephone interview and to schedule a convenient time to complete the survey over the phone. All participants were sent appointment reminders via text message before the interview. We approached 208 eligible mothers, of whom 8 refused to participate (response rate = 96.2%). Therefore, the final sample size comprised 200 Syrian refugee women.

At the beginning of the telephone interview, the research assistant asked the mother if she was in a private place and if she felt comfortable starting the interview without interruption. During the interview, the mothers’ responses were entered into Qualtrics XM (Qualtrics, Seattle, WA), a secure, password-protected, web-based software. The questionnaires took approximately 20–30 min to complete. After completing the questionnaires and after each interview, mothers were provided an electronic voucher ($10).

### Measures

#### Postpartum depression symptoms

The Edinburgh Postnatal Depression Scale (EPDS) was used to measure postpartum depression symptoms (22). It consists of 10 items rated on a 4-point Likert scale (0 to 3) to represent the duration or severity of depression symptoms. Item scores are summed, and the total score ranges from 0 to 30, indicating the severity of depression, with higher scores reflecting greater severity. For the Arabic version, a cutoff score of 10 has a sensitivity of 91% and a specificity of 84% (23). Cronbach’s alpha in this study was 0.92.

#### Social support

The Multidimensional Scale of Perceived Social Support (MSPSS-AW) was used to measure social support (22–25). It consists of 12 items rated on a 3-point scale of 1 = *disagree*, 4 = *neutral,* and 7 = *agree* (23, 25). It includes three subscales, each with four items assessing perceived support from family, friends, and husband. The total score ranges from 12 to 84. The scale demonstrated construct validity (23, 25). Cronbach’s alpha was 0.89 in this study.

#### Resilience

The Resilience Scale (CD-RISC) was used to measure psychological resilience (26). The CD-RISC consists of 25 items rated on a 5-point Likert scale (0 = *not true at all*, 4 = *often true*). The total score is obtained by summing item scores ranging from 0 to 100, with higher scores representing a greater level of resilience. The CD-RISC demonstrated reliability and construct validity among individuals with PTSD (26). The Arabic version of the CD-RISC showed excellent internal consistency in this study (*α* = 0.91).

#### Posttraumatic stress disorder (PTSD) symptoms

The PTSD Checklist for DSM-5 (PCL-5) was used to measure PTSD symptoms (27). The PCL-5 is a self-report measure including 20 items rated on a 5-point scale ranging from *not at all* (0) to *extremely* (4). The total score obtained by summing the item responses ranges from 0 to 80, with higher scores indicating greater symptom severity. The PCL-5 has excellent internal consistency (*α* = 0.90) and convergent validity (28). The Arabic version showed adequate convergent validity with a sensitivity of 0.82 and a specificity of 0.70 (29). Cronbach’s alpha was 0.96 in this study.

#### Anxiety

The Generalized Anxiety Disorder-7 (GAD-7) measures anxiety symptoms (30). The GAD-7 is a 7-item scale rated on a 4-point Likert scale ranging from *not at all* (0) to *nearly every day* (3). The total score is a sum of all items and ranges from 0 to 21. Higher scores represent greater anxiety severity. The validated Arabic version of the GAD-7 had a Cronbach’s alpha of 0.76 (31); it was 0.95 in this study.

#### Intimate partner violence

The Abuse Assessment Screen (AAS) is a 5-item screening tool to assess abuse perpetrated against women, particularly pregnant women (32). Each item is dichotomous with *yes* or *no* answers to questions about emotional, physical, and sexual violence. A positive answer to any question indicates abuse. Compared with the provider interview, the AAS has 93–94% sensitivity and 55–99% specificity and demonstrates a test–retest reliability of 0.91 (33). The AAS was administered to assess intimate partner violence across different cultures (33), including refugee women in Jordan (34).

#### Postmigration stressors

The Postmigration Living Difficulties Scale (PMLD) measures postmigration issues faced by refugees over the past 12 months (35). It comprises 17 items, each rated on a 5-point scale *from not a problem* (1) to *a very serious problem* (5). The total scale score is obtained by summing all item responses, yielding a range from 0 to 68, with higher scores indicating higher severity of postmigration stressors. The Arabic version of the PMDL, validated among Syrian refugees in Türkiye, demonstrated good internal consistency reliability (*α* = 0.76) (36). In this study, Cronbach’s alpha was 0.70.

#### Sociodemographic and clinical characteristics

We collected information on the participants’ socio-demographic characteristics (i.e., age, marital status, level of education, employment status, income adequacy, receipt of financial aid, and length of residence in Türkiye) and clinical characteristics (i.e., number of pregnancies, number of children, gestational age at birth, mode of birth, infant’s birth weight, gender of the infant, type of infant’s feeding, high-risk pregnancy [medical or obstetrical complications, e.g., preeclampsia, diabetes mellitus], lifetime history of depression or anxiety, and perceived unmet mental health treatment need [“Have you felt that you needed mental health treatment for postpartum depression but you did not get it?”]).

### Data analysis

Descriptive statistics (i.e., means, standard deviation, and frequencies) were used to describe the sample sociodemographic and clinical characteristics and the key study variables (e.g., postmigration stress, intimate partner violence, and PPD). The normality of the data was checked using the Kolmogorov–Smirnov test. The dependent variable, EPDS, was scored as a categorical variable in the study: depression symptoms (EPDS ≥ 10) vs. no depression symptoms (EPDS < 9), as it did not meet the normality assumption. We first conducted bivariate analyses to examine the association between the outcome variable (PPD) and the participants’ characteristics. Chi-square or Mann–Whitney U tests were used to examine differences in postpartum depression symptoms by participants’ characteristics. Spearman rank correlations were used to examine the associations among continuous variables. Next, logistic regression was conducted to examine the psychological predictors of PPD, controlling for significant covariates identified in the bivariate analyses. The results of the logistic regression then provided preliminary evidence of relationships among the study variables, which informed the selection of variables included in the exploratory path model analysis (i.e., postmigration stress, social support, resilience, and PPD). While this study initially aimed to examine the indirect pathways between IPV and PPD, IPV was excluded from the path analysis due to a low report rate and a non-significant association with PPD in logistic regression.

The indirect effect was examined using the weighted least squares mean and variance adjusted (WLSMV) estimator, which is appropriate for categorical dependent variables. We used a top-down approach by including covariates based on bivariate analysis in the path model, removing the nonsignificant covariates from the path model one at a time while assessing the improvement in model fit (37). The bootstrapping technique with bias-corrected confidence interval estimates was conducted to examine significant indirect pathways, i.e., social support and resilience. The hypothesized model was evaluated for goodness of fit using the following measures: Chi-square goodness of statistic (*p* > 0.05), Comparative Fit Index (CFI ≥ 0.90), Tucker-Lewis Index (TLI ≥ 0.90), Root Mean Square Approximation (RMSEA ≤ 0.08), and standardized root mean square residual (SRMR < 0.08). The standardized coefficients were reported. The analyses were conducted using IBM SPSS version 28 and M*plus* version 8.0 (38). Alpha was set *a priori* at 0.05 for all statistical tests.

## Results

### Characteristics of participants

Participants’ sociodemographic and clinical characteristics are summarized in Table 1. More than half of the participants were aged 27 years or older (51.0%), unemployed (99.0%), had less than a high school education (81.5%), and reported insufficient income (88.0%). Most were married (99.5%), multiparous (94.5%), and gave birth vaginally (66.0%), with 34.5% having experienced high-risk pregnancies. Only 3% of mothers reported intimate partner violence, while 25% had unmet mental health treatment needs. PPD symptoms were significantly differentiated by parity, high-risk pregnancy, history of depression, and unmet mental health treatment needs for PPD.

**Table 1:** Participants characteristics by the presence of PPD in Syrian refugee women (*N* = 200).

Characteristics	Total sample *N* (%)	With PPD *n* (%)	Without PPD *n* (%)	$x^{2}$	*p*-value
Age					
18–26	98 (49.0)	17 (51.5)	81 (48.5)	0.251	0.882
27–35	79 (39.5)	13 (39.4)	66 (39.5)		
36–44	23 (11.5)	3 (9.1)	20 (12.0)		
Education level					
Less than high school	163 (81.5)	24 (72.7)	139 (83.2)	4.764	0.092
High school	20 (10.0)	3 (9.1)	17 (10.2)		
Some college/college	17 (8.5)	6 (18.2)	11 (6.6)		
Employment status					
Unemployed	198 (99.0)	31 (93.9)	167 (100.0)	10.223	0.027^a^
Part-time employed	2 (1.0)	2 (6.1)	0 (0.0)		
Marital status					
Married	199 (99.5)	33 (100.0)	166 (99.4)	0.199	1.00
Divorced	1 (0.5)	0 (0.0)	1 (0.6)		
Perception of income adequacy					
Not adequate at all	61 (30.5)	13 (39.4)	48 (28.7)	1.584	0.453
Inadequate	115 (57.5)	16 (48.5)	99 (59.3)		
Adequate	24 (12.0)	4 (12.1)	20 (12.0)		
Length of stay in Türkiye (years)					
1–4	20 (10.0)	5 (15.2)	15 (9.0)	1.338	0.512
5–8	129 (64.5)	21 (63.6)	108 (64.7)		
9 or more	51 (25.5)	7 (21.2)	44 (26.3)		
Planned pregnancy					
Yes	68 (34.0)	8 (24.2)	60 (35.9)	1.677	0.195
No	132 (66.0)	25 (75.8)	107 (64.1)		
High risk pregnancy					
Yes	69 (34.5)	18 (54.5)	51 (30.5)	7.027	0.008
No	131 (65.5)	15 (45.5)	116 (69.5)		
Para					
Primi	11 (5.5)	5 (15.2)	6 (3.6)	7.083	0.008
Multi	189 (94.5)	28 (84.8)	161 (96.4)		
Infant age (months)					
1–4	23 (11.5)	2 (6.1)	21 (12.6)	1.250	0.535
5–8	53 (26.5)	10 (30.3)	43 (25.7)		
9–12	124 (62.0)	21 (63.6)	103 (61.7)		
Mode of delivery					
Vaginal delivery	124 (62.0)	22 (66.7)	102 (61.1)	0.365	0.546
Cesarean section	76 (38.0)	11 (33.3)	65 (38.9)		
Number of living children					
1–2	61 (30.5)	11 (33.3)	50 (29.9)	1.186	0.553
3–4	101 (50.5)	14 (42.4)	87 (52.1)		
5 or more	38 (19.0)	8 (24.2)	30 (18.0)		
Feeding method					
Breastfeeding	114 (57.0)	20 (60.6)	94 (56.3)	0.695	0.706
Bottle feeding	45 (22.5)	8 (24.2)	37 (22.2)		
Both	41 (20.5)	5 (15.2)	36 (21.6)		
Lifetime diagnosis of depression or anxiety					
Yes	14 (7.0)	8 (24.2)	6 (3.6)	9.381	0.002
No	186 (93.0)	25 (75.8)	161 (96.4)		
Unmet mental health treatment for PPD					
Yes	50 (25.0)	21 (63.6)	29 (17.4)	31.464	<0.001
No	150 (75.0)	12 (36.4)	138 (82.6)		
Intimate partner violence					
Yes	6 (3.0)	3 (9.1)	3 (1.8)	5.038	0.058^a^
No	194 (97.0)	30 (90.9)	164 (98.2)		

PPD = Postpartum depression. Participants with PPD had a score of 10 or above on the Edinburgh Postnatal Depression Scale.

^a^Fisher’s exact test.

### Bivariate relationships

PPD was associated with all main variables (Table 2). Post-migration stress was associated with resilience (*r* = −0.229, *p* < 0.001) and social support (*r* = −0.193, *p* < 0.001). However, social support was not associated with resilience (*r* = 0.137, *p* = 0.052).

**Table 2:** Mental health characteristics of Syrian refugee women by the presence of PPD (*N* = 200).

Characteristics	Total sample Mean (SD)	With depression Mean (SD)	Without depression Mean (SD)	Z-test	*p*
Resilience	61.4 (12.6)	47.5 (12.8)	64.1 (10.6)	−6.146	<0.001
Posttraumatic stress	2.0 (7.1)	4.4 (9.9)	1.6 (6.4)	3.998	<0.001
Social support	54.6 (22.5)	44.1 (24.2)	56.6 (21.6)	−2.860	0.004
Anxiety	3.7 (5.1)	11.3 (5.2)	2.2 (3.5)	7.974	<0.001
Postmigration stress	13.2 (7.4)	16.5 (9.0)	12.5 (6.8)	2.552	0.011

PPD = Postpartum depression. Participants with PPD had a score of 10 or above on the Edinburgh Postnatal Depression Scale.

### Predictors and indirect associations with PPD

Regression analysis revealed that, after controlling for other variables in the model, the odds of PPD increased significantly by 33% for each one-unit increase in the anxiety score (Adjusted Odds Ratio [AOR]: 1.33; 95% CI: 1.17–1.53) (Table 3). Conversely, for each one-unit increase in the resilience score, the odds of PPD decreased significantly by 8% (AOR: 0.92; 95% CI: 0.867–0.979).

**Table 3:** Psychological predictors of postpartum depression in Syrian refugee women settling in Türkiye.

Predictor	Unadjusted OR	95% CI	*p*	Adjusted OR	95% CI	*p*
Resilience	0.888	0.853–0.925	<0.001	0.921	0.867–0.979	0.008
Social support	0.975	0.959–0.992	0.004	0.993	0.965–1.02	0.617
Anxiety	1.405	1.275–1.549	<0.001	1.338	1.17–1.53	<0.001
Posttraumatic stress	1.041	0.999–1.085	0.058	0.978	0.92–1.04	0.48
Postmigration stress	1.071	1.020–1.125	0.006	1.027	0.94–1.12	0.561
Intimate partner violence	5.467	1.053–28.378	0.043	2.972	0.19–47.11	0.440

Adjusted odds ratio controlled for parity, high-risk pregnancy, lifetime diagnosis of depression or anxiety, and unmet need for mental health treatment.

**Figure 1:**
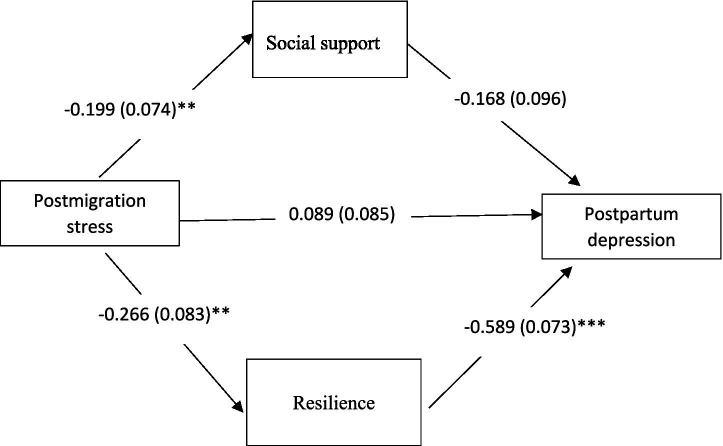
The path model with standardized probit coefficients for the direct effects. Model adjusted by parity. **p < 0.01; ***p < 0.001.

Figure 1 displays the path diagram for the standardized probit coefficients. The model adjusting for parity fit the data well, X^2^ (2, *N* = 200) = 2.798, *p* = 0.247; CFI = 0.99; TLI = 0.94; RMSEA = 0.045[90% CI: 0.000–0.155]; SRMR = 0.056. In the model, there was a direct effect from postmigration stress to resilience (*β* = −0.266, *p <* 0.01) and social support (*β* = −0.199, *p* < 0.01). In addition, there was a significant direct effect from resilience (*β* = −0.589, *p* < 0.001) to PPD. Path analysis indicated that social support (*β* = 0.046, 95% CI: 0.012–0.108) and resilience (*β* = 0.163, 95% CI: 0.075–0.25) indirectly affect the relationship between postmigration stress and PPD (Table 4).

**Table 4:** Standardized probit coefficients for the indirect effects of postmigration stress on PPD via social support and resilience.

Path	Standardized coefficients	SE	95% CI
Postmigration stress → Social support → PPD	0.033	0.024	0.004–0.079
Postmigration stress → Resilience → PPD	0.157	0.053	0.077–0.244
Sum of the indirect effect	0.190	0.058	0.098–0.286

PPD = Postpartum depression.

## Discussion

This study was the first to explore psychological predictors and indirect pathways with PPD in Syrian refugee women settling in Türkiye. The findings identified anxiety and resilience as significant predictors of PPD in this population. There is strong evidence on the role of anxiety in predicting PPD (1, 4). This finding among Syrian refugee women is noteworthy because if healthcare providers screen for anxiety symptoms in this population, they could provide early interventions to help prevent the onset or severity of PPD. The timely interventions may help reduce the treatment gap for PPD in this vulnerable population. Another important predictor of PPD in Syrian refugee women is resilience. This is consistent with the existing literature which indicates an association between resilience resources and fewer symptoms of PPD (39). An integrative review highlighted the crucial role of resilience in preventing the development of PPD in immigrant and refugee women, emphasizing their ability to maintain hope and navigate life challenges (40).

Other psychological factors examined in this study that were not related to PPD in refugee women include postmigration stress, intimate partner violence, post-traumatic stress disorder, and social support. This contrasts with previous research (4, 41), which has identified postmigration stress and PTSD as risk factors for PPD. One possible explanation is that after adjusting for other psychological variables and covariates, the impact of postmigration stress and PTSD may have been moderated or mediated by additional factors unique to Syrian refugee women in Türkiye. For example, Acar et al. (36) found that postmigration stress in Syrian refugee adults who settled in Türkiye was positively associated with postmigration growth, defined as a positive psychological change experienced as an outcome of highly stressful life circumstances (42). Hence, a deeper understanding of how postmigration stress and post-traumatic stress could contribute to PPD in Syrian refugee women is required. This is crucial because these women might feel reluctant to report specific traumatic events related to migration due to the social desirability effect. This was also reflected in the fact that only 3% of our sample reported intimate partner violence, which was not related to PPD in the regression model and could be related to underreporting of this issue and sample or measurement biases. It is noteworthy that a qualitative study of Syrian refugee women living in Canada revealed that these women are dependent on their partners as the primary source of social support (14). However, further mixed-methods research is needed to understand spousal support in refugees.

Although social support was related to PPD, it did not predict PPD after controlling for other variables in the model. This suggests that social support is a challenging experience for immigrant and refugee women (40). Previous research on Syrian refugee women settled in Türkiye showed that women’s perceived social support decreased as their length of stay in Türkiye increased, whereas their hopelessness level rose (43). Given that most participants in our study had lived in Türkiye for at least 5 years, this trend may partially explain the limited influence of social support on PPD in our sample. While the intersectionality of culture, gender, and migration policy indicates the importance of family as a source of social support for immigrant and refugee women, migration policies could contribute to the fragmentation of the family unit and undervalue cultural traditions necessary to provide the appropriate social support for these women (40, 44). This is crucial as our study indicated that postmigration stress and PPD were significantly indirectly associated through social support. Congruently, prior evidence showed a significant correlation between low levels of social support and PPD in refugee women (1, 4).

Furthermore, our findings showed an indirect path between postmigration stress and PPD through resilience in refugee women. Notably, resilience plays a salient role in the vulnerability of PPD (40). A qualitative study of 30 immigrant and refugee women revealed that despite numerous traumatic events and life challenges, resilience framed the experiences of these women as they had the opportunity to grow stronger while maintaining a sense of control over their life situation (44). Accordingly, healthcare providers need to create strategies and tailor interventions that facilitate resilience in Syrian refugee women and ultimately mitigate the risk of PPD.

### Limitation

This study has some limitations. First, we employed convenience sampling to recruit Syrian refugee women via referrals from a single NGO, RASASA, in Istanbul. Although this method was selected to provide direct access to refugee women, recruitment was limited to those connected to RASASA and who voluntarily agreed to participate. Therefore, the sample may not represent the broader population of Syrian refugee women. Second, there may be other variables influencing PPD that were not examined or included in the model. Third, a cross-sectional research design was used in the current study, which may preclude causal or temporal inferences Thus, longitudinal research is needed to further test the path model explored in the current study. Fourth, while collecting data via telephone-based interviews is a reliable and accessible method (45), underreporting is still a concern due to the social desirability effect, as refugee women might feel hesitant to disclose issues related to their mental health, such as posttraumatic stress and intimate partner violence. Hence, further studies are required to focus on these critical issues, considering the limited information available on Syrian refugee women in Türkiye. Finally, whereas the path model had strong fit indices, there is potential overfitting due to the exploratory nature of the analysis and the sample characteristics. Therefore, to validate the indirect pathways, confirmatory designs and representative samples are recommended in future studies.

### Conclusions and implications

This study revealed that anxiety and resilience are important predictors of PPD in Syrian refugee women. Our findings also suggest indirect associations between postmigration stress and PPD through resilience and social support. Hence, clinical and policy initiatives are recommended to address mental health in refugee women through screening programs integrated in perinatal health care in both hospital and community-based settings. In addition, interventions that promote social support and resilience in Syrian refugee women may be beneficial in improving their PPD symptoms. Specifically, understanding the needs of refugee women and the intersection of their culture, gender roles, and migration policies is crucial to planning and implementing prevention interventions that enhance coping and resilience capacity. This is crucial as building social support and connections among refugee women should also be advocated (19). Finally, while our study is cross-sectional and exploratory, future community-based participatory research that involves collaboration between researchers and community stakeholders (46) serving refugee women is needed to better understand refugee women’s mental health and address their needs through action steps.

## Data Availability

The original contributions presented in the study are included in the article/supplementary material, further inquiries can be directed to the corresponding author.
